# RNA-Seq Analyses Identify Frequent Allele Specific Expression and No Evidence of Genomic Imprinting in Specific Embryonic Tissues of Chicken

**DOI:** 10.1038/s41598-017-12179-9

**Published:** 2017-09-20

**Authors:** Zhu Zhuo, Susan J. Lamont, Behnam Abasht

**Affiliations:** 1 Department of Animal and Food Sciences, University of Delaware, Newark, Delaware United States; 2 Department of Animal Science, Iowa State University, Ames, Iowa United States

## Abstract

Epigenetic and genetic *cis*-regulatory elements in diploid organisms may cause allele specific expression (ASE) – unequal expression of the two chromosomal gene copies. Genomic imprinting is an intriguing type of ASE in which some genes are expressed monoallelically from either the paternal allele or maternal allele as a result of epigenetic modifications. Imprinted genes have been identified in several animal species and are frequently associated with embryonic development and growth. Whether genomic imprinting exists in chickens remains debatable, as previous studies have reported conflicting evidence. Albeit no genomic imprinting has been reported in the chicken embryo as a whole, we interrogated the existence or absence of genomic imprinting in the 12-day-old chicken embryonic brain and liver by examining ASE in F1 reciprocal crosses of two highly inbred chicken lines (Fayoumi and Leghorn). We identified 5197 and 4638 ASE SNPs, corresponding to 18.3% and 17.3% of the genes with a detectable expression in the embryonic brain and liver, respectively. There was no evidence detected of genomic imprinting in 12-day-old embryonic brain and liver. While ruling out the possibility of imprinted Z-chromosome inactivation, our results indicated that Z-linked gene expression is partially compensated between sexes in chickens.

## Introduction

Expression difference at the allelic level, termed allele specific expression (ASE), is caused by *cis*-regulatory elements, such as *cis*-acting DNA sequence variants, epigenetic marks, and post-transcriptional modifications. Previous studies identified ASE as a frequent event in mammals. More than 50% and 30% of genes show ASE patterns in humans and mice, respectively^1–6^. In agricultural animals, ASE genes have been found related to economically important traits^7–9^. Particularly, ASE SNPs have been observed in response to Marek’s disease (MD) virus in chickens^10,11^, and selection using those ASE SNPs reduced MD incidence after one generation of selection^12^. Thus, finding ASE is critical for establishing the connection between genotype and phenotype and for application in agriculture.

A unique and intriguing type of ASE is caused by genomic imprinting, in which autosomal genes are monoallelically expressed from either the paternal or maternal allele. Divergent statuses of epigenetic modification between the parental alleles of imprinted genes cause the gene expression to be turned “on” or “off” according to their parental origin. Although the majority of genes are expressed from both parental alleles, a small portion of genes has been classified as imprinted in mammals and these genes are often associated with embryonic growth and development. In agricultural animals, imprinted genes have been identified in cows, pigs and sheep (for references such as^13–15^ and reviewed by^16^); however, the evidence regarding existence of genomic imprinting in chickens is conflicting. It has been reported that *IGF2* is imprinted in some chicken embryos and the expressed allele can be of either paternal or maternal origin^17^, but several other studies maintained that *IGF2* is biallelically expressed^18–20^. Additionally, the chicken orthologs of imprinted genes in mammals, such as *INS*, *ASCL2/CASH4*, *UBE3A*, *Dlk1*, *GATM*, and *M6P/IGF2R*, were found biallelically expressed in chickens^19–23^. Recently, several genome-wide investigations of genomic imprinting in chickens using RNA-Seq have been conducted. Fresard *et al*. reported that genomic imprinting is absent in the 4.5-day-old chicken embryos^24^. Wang *et al*. focused on studying brains from day-old chickens posthatch and also didn’t identify evidence of genomic imprinting^25^. In contrast, Pinto *et al*. recently reported finding thousands of SNPs with parent-of-origin effect in chicken hypothalamus, liver and breast muscle at 56 days of age^26^. Collectively, even though most studies indicated the absence of genomic imprinting in chickens, additional critical examination of different tissue types and developmental stages is necessary to make a conclusive argument.

Here, we performed a genome-wide investigation of ASE using chicken liver and brain samples at embryonic day 12 from a reciprocal cross system. Our main objective was to survey genomic imprinting to answer the question of whether genomic imprinting exists in chickens. We also examined gene expression on the Z chromosome to study dosage compensation in chickens.

## Materials and Methods

### Sample Collection

We utilized two highly inbred experimental chicken lines, Leghorn (*Ghs13*) and Fayoumi (*M5*.*1*), that are maintained at Iowa State University. Eggs from Leghorn × Fayoumi cross (LF) and from Fayoumi × Leghorn cross (FL), as well as Fayoumi and Leghorn lines, were collected and kept in an egg cooler at Iowa State University until air-shipped to the University of Delaware. For clarity, F1 cross egg samples were named as paternal origin followed by maternal origin. The eggs were incubated in an egg incubator at 100 °F, 70% humidity. At day 10, the fertility of each egg was checked by candling, and non-fertile eggs were properly disposed. At day 12, brain, liver and a few other tissues were harvested from the embryos and the tissue samples were immediately frozen in liquid nitrogen and stored in a −80 °C freezer. For the purposes of PCR-based sexing and DNA-Seq, the remainder of each embryo was also frozen and preserved. All animal protocols for production of the fertile eggs were conducted with the approval of Iowa State University IACUC Log #4-03-5425-G. No approval of University of Delaware AACUC was required for chicken embryo experiments.

### PCR-based Sexing

Genomic DNA was isolated from ~25 mg of each embryo using a DNeasy Blood & Tissue Kit (Qiagen). A PCR-based method adapted from Clinton *et al*. was used to determine the sex of each embryo^27^. Two pairs of primers were used to separately amplify a DNA fragment on the W chromosome of female chickens, *XhoI*, and a DNA fragment on ribosome 18 S RNA gene as positive control. The sexes of the embryos were determined based on the gel electrophoresis of PCR products (Table S1 and Figure S1).

### DNA-Seq

The genomic DNA from inbred Fayoumi and Leghorn chickens was sequenced to serve two purposes: first, to identify the DNA polymorphisms that could discriminate the parental origin of alleles in the F1 reciprocal crosses, and second, to create a customized reference genome with parental SNPs masked to reduce reference bias. Two pooled samples were generated by mixing equal molar amounts of genomic DNA from 14 Fayoumi embryos (7 females and 7 males) and 14 Leghorn embryos (7 females and 7 males), separately, and sent to the genomics core facility at Michigan State University for library preparation and sequencing. TruSeq DNA Library Preparation Kit LT (Illumina) was used and the resultant libraries were sequenced on 2 lanes on llumina HiSeq 2500 system with Rapid Run flow cell (v1) using a 150-cycle paired-end sequencing protocol.

The quality of the sequencing data was examined using FastQC v0.11.2^28^. The reads were mapped to the chicken reference genome Galgal 4 (Ensembl) using the default setting of BWA mem v0.7.12^29,30^. Unplaced scaffolds of the reference genome sequence were discarded before read alignment. The SNPs of Fayoumi and Leghorn genomes were identified following the best practice of Genome Analysis ToolKit (GATK) v3.3 with default parameters^31–33^. Briefly, the alignment BAM file from each lane was sorted according to chromosome coordinates, and the duplicated reads were marked for exclusion later in the variant-calling step. Realignment was performed around indels, and then the base quality was recalibrated to reduce errors produced by the sequencing machine. The BAM files were merged for each sample and processed again through marking duplication, realignment and recalibration. The variants from Fayoumi and Leghorn were identified separately using HaplotypeCaller in GVCF mode and outputted jointly using GenotypeGVCFs. The SNPs were extracted, and an additional criterion of read depth (DP) greater than 10 was applied using custom python scripts. To account for sequencing error, a heterozygous genotype call was reassigned as homozygous when the allele count was less than DP * 1%. A customized reference genome with Fayoumi and Leghorn SNPs replaced by “N” was generated using GATK FastaAlternateReferenceMaker for later RNA-Seq analysis. A list of loci in which both Fayoumi and Leghorn are homozygous but carrying different alleles was generated. At those loci, the genotypes of F1 crosses were predicted to be heterozygous.

### RNA-Seq

We chose 12 male embryonic samples (2 Fayoumi, 2 Leghorn, 4 FL and 4 LF) for RNA sequencing. Total RNA from brain and liver tissues was extracted using mirVana miRNA Isolation Kit (Thermo Fisher Scientific). The quality of RNA samples was assessed using a Bioanalyzer 2100 (Agilent Technologies), and the RNA integrity numbers (RINs) for all samples were greater than 9.8. Stranded cDNA libraries were prepared using a TruSeq Stranded Total RNA Library Prep Kit (Illumina, Inc.). During library preparation, cDNA fragments from each sample were ligated with a unique index adapter for further discrimination. The cDNA libraries were validated by Bioanalyzer 2100 analysis (Agilent) and then normalized to 10 nM using Tris buffer (Tris-Cl 10 mM, 0.1% Tween 20, pH 8.5). Ten ul from each library were pooled into a single sample and sequenced on an Illumina HiSeq. 2000 system using a 75-cycle paired-end sequencing protocol.

The RNA-seq data from each sample was demultiplexed utilizing the unique index adapters. A quality check of the RNA-Seq data was performed using FastQC (v0.11.2)^28^. The reads were mapped to the customized chicken reference genome using the STAR (v2.4.1c) 2-pass method with default parameters^34^. Variants relative to the chicken reference genome were discovered using GATK^31–33^. The workflow for variant calling from RNA-Seq data is generally similar to that for DNA-Seq as previously described, except that reads were split to remove “N”s between exon segments and variant calling was conducted using HaplotyperCaller in variant discovery mode with all samples. Low quality calls (QD < 2), or variants with strong strand bias (FS > 30) and SNP clusters (3 SNPs in 35 bp window) were excluded from further analysis. The resultant VCF files were further partitioned to obtain SNPs for each individual sample. Meanwhile, additional filters, including read depth (DP < 10) and genotype quality (GQ < 30), were imposed to obtain high confidence “genotypes”. An allelic read count less than DP * 1% was considered as a sequencing error and, therefore, was reassigned as 0.

### Detection of ASE and Genomic Imprinting

We performed both within samples analysis and meta-analysis across multiple samples to detect ASE. The loci where the predicted genotypes for F1 crosses (based on the genotypes of parental lines) were heterozygous and the SNPs were identified through RNA-Seq were designated as testable. Monoallelic expression at testable loci was considered as ASE. If the expression was biallelical, a binomial test was performed on the allele read counts in F1 crosses to test whether the allelic expression was deviated from equal expression. P-values were adjusted using Benjamini-Hochberg method in R, and an adjusted p-value cutoff of 0.1 was applied to claim statistical significance of the ASE SNPs for each sample. The allele with higher expression is referred to as the preferred allele. Additionally, a meta-analysis on samples with the same preferred allele was carried out using Fisher’s combining probability test to assess the ASE across the biological replicates^35^. Tissue-specific ASE was evaluated between brain and liver. The genes identified as ASE in one tissue type but not the other were defined as tissue-specific ASE. Of note, when a SNP (ASE or not) was detected using RNA-seq data in two or more samples of the same tissue, the SNP-containing gene was declared as being expressed in that type of tissue. SnpEff ^36^ was used to annotate the ASE SNPs and to identify the ASE genes.

For the ASE SNPs identified from within sample analysis, a parental gene expression ratio (read count of paternal allele or maternal allele/total read count) was calculated. Genomic imprinting was evaluated based on whether the parental origin of the preferred allele is the same in reciprocal crosses. A putative imprinted gene was identified if an ASE SNP was detected for the gene in at least two samples in each reciprocal cross, and the parental origin of the preferred allele was the same in all samples with the detected ASE SNP.

### Dosage Compensation Analysis

To study dosage compensation between autosomes and sex chromosomes, the gene expression levels in fragments per kilobase of transcript per million mapped reads (FPKM) were analyzed for each sample using Cufflinks (v2.1.1)^37^ after STAR (v2.4.1c) alignment. The FPKM values greater than 0.1 were Log2 transformed and the mean FPKM of Z chromosome and autosomes were calculated for each sample to obtain the difference of gene expression between Z and A by subtracting, i.e., Δ_Z-A_ = Mean Log2(FPKM)_*Z_genes*_ – Mean Log2(FPKM)_*A_genes*_^38^. Then, the mean expression difference between Z and A for each tissue type was obtained by averaging Δ_Z-A_ values of all samples from the same tissue type. Equal expression between Z chromosome and autosomes would result in the value 0.

### Verification of ASE Genes

Four ASE genes in brain and 6 ASE genes in liver were selected and further verified using Sanger sequencing. The genes were chosen to represent different average expression ratios, depths of RNA-Seq reads, and locations of ASE SNPs. One sample from each F1 cross was randomly selected to perform the verification experiments. PCR primers were designed using Primer 3^39^ to amplify the sequences containing target ASE SNPs and for Sanger Sequencing. Because the genotypes of F1 crosses were established according to the DNA-Seq data from pooled genomic DNA, we sequenced genomic DNA at target SNP loci to confirm the predicted genotypes. Due to a deletion located upstream of the target SNP for an unnamed gene (ENSGALG00000027334), the sequencing trace showed double peaks at the target locus. The genotype of this unnamed gene was verified by amplification-refractory mutation system (ARMS) PCR^40^ (data not shown). Total RNA was treated with DNA-free DNA Removal Kit (Thermo Fisher Scientific) to eliminate genomic DNA. First-strand cDNA was synthesized using SuperScript IV First-Strand Synthesis System and Oligo (dT) primers (ThermoFisher Scientific) from the same RNA samples used for RNA-Seq library preparation. PCR of genomic DNA and cDNA was carried out using AccuPrime *Pfx* SuperMix (Thermo Fisher Scientific). The expression ratio between the two alleles were estimated using Minor Variant Finder (Thermo Fisher Scientific) and ImageJ^41^ based on the sequencing traces of cDNA from the F1 samples. Genomic DNA of a Fayoumi sample was also sequenced at the targeted regions to provide a homozygous control to facilitate detection of variants when using Minor Variant Finder. The chromatograms of Sanger sequencing were viewed using SnapGene software (GSL Biotech).

### Data Availability

The datasets generated during and/or analysed during the current study are available in the NCBI Sequence Read Archive (Accession number SRP102082). The ASE SNPs identified in this study are included in the Supplemental datasets.

## Results and Discussion

### DNA-Seq of Inbred Parental Lines

A total of 99 GB high-quality sequencing data, consisting of 155.8 million and 137.4 million 150b sequence reads, was generated by sequencing DNA pools from Fayoumi and Leghorn samples, respectively. The estimated mean sequencing coverage was 21.79× for Fayoumi and 19.22× for Leghorn (Table S2). Around 92% of the sequence reads were properly paired and mapped to the chicken reference genome (Ensembl Galgal 4.0). We identified 5,072,830 and 4,632,414 variants - of which 4,442,390 and 4,055,489 were single nucleotide variations - from Fayoumi and Leghorn data, respectively. At 93.6% and 95.8% of the detected SNP loci, Fayoumi and Leghorn lines were homozygous, respectively. Corroborated with a previously reported DNA re-sequencing study using the same chicken lines^42^, the results suggested that the parental chicken lines used in the present study are highly homozygous across the genome. Importantly, at 3,071,441 loci the two inbred parental lines are homozygous for different alleles, which generate unambiguous heterozygous genotypes in F1 offspring and makes it possible to distinguish the parental origin of the alleles. Those loci were further verified to identify the corresponding genes, which included up to 83.2% of the annotated genes (Galgal4.78).

### RNA-Seq Analysis with Customized Genome

Sequence read alignments to a reference genome are prone to bias, because reads carrying a reference allele have a slightly better chance of being mapped to the reference genome than the reads carrying an alternative allele. To minimize this so-called “reference bias” in our RNA-seq reads alignment [42–44], a customized reference genome was generated by masking the SNP loci from Fayoumi and Leghorn DNA-seq data. A total of 1.55 billion reads were obtained from 305 GB of RNA-Seq data, averaging 65 million reads per sample. The reads were mapped to the chicken reference genome Galgal 4 (Ensembl, original reference genome) as well as the customized reference genome. On average, 85% of the reads were mapped, with mapping rates from the customized reference genome being slightly higher than that of the original reference genome (Table S3).

**Figure 1:**
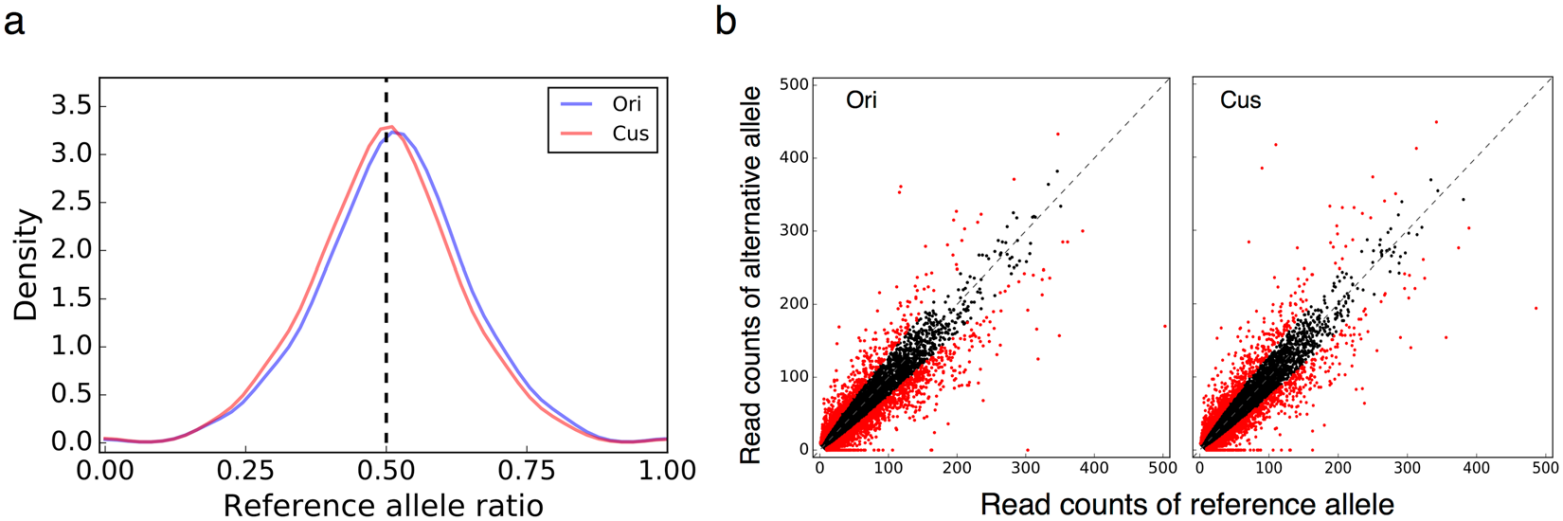
Using a customized reference genome reduced reference bias: (**a**) The distribution of the reference allele ratio with the original reference genome and customized reference genome. (**b**) The scatterplot of read counts of reference allele and alternative allele at SNP positions from results of original reference genome and customized reference genome. ASE SNPs are highlighted in red.

With the customized reference genome, the average reference ratio in the F1 cross samples was reduced from 50.98% to 49.66%. Thus, a new bias towards alternative alleles was generated after masking the reference genome. To achieve a more accurate estimation of allelic expression, Wang *et al*. proposed to average the read counts obtained using both the original and the customized reference genomes^43^. However, in our case, the data had greater bias towards the reference allele with the original reference genome than the bias towards the alternative allele with the customized reference genome. Consequently, the reference allele ratio after averaging read counts was 50.78%, which was more biased than that from the customized reference genome. Therefore, we conducted further analyses based on the results from the customized reference genome. It is evident that the results from the customized reference genome had an improved distribution of the reference allele ratio, compared with the right-shifted distribution of the original reference genome (Fig. 1a). The allelic read counts for reference allele and alternative allele at heterozygous loci were more evenly dispersed along the diagonal with the customized genome (Fig. 1b). In conclusion, using the customized reference genome with SNPs masked instead of the original reference genome is an effective method to reduce reference bias.

### SNPs Identified in RNA-Seq and the Consistency Between Predicted Genotypes and RNA Expression

Considering the chicken lines used in the present study are highly inbred, we expected that the DNA-Seq data from the parental genomic DNA pool of 14 individuals could be used to establish high-fidelity predicted genotypes for the F1 crosses. To confirm, we examined the consistency of genotypes between DNA-Seq- and RNA-Seq-based genotype calls. We made two comparisons. First, we compared the DNA-Seq- and RNA-Seq-based genotype calls for inbred lines. On average, 93,137 SNPs and 55,675 SNPs were identified in Fayoumi brain and liver RNA-Seq data; 109,631 SNPs and 71,889 SNPs were identified in Leghorn brain and liver RNA-Seq data, respectively. Of those SNP loci from RNA-Seq data, about 15% were not identified in DNA-Seq data, but the SNP loci identified in both datasets showed 98.6% consistency of genotypes. Second, we compared the predicted genotypes of F1 embryos based on the DNA-Seq data with their observed genotype calls based on RNA-seq data. The numbers of SNPs identified in FL cross versus LF cross did not show a significant difference. On average, 133,834 SNPs and 79,260 SNPs were identified in the brain and liver tissues, respectively. Likewise, 99.75% of the SNP loci found in both datasets showed consistency of genotypes. Except for technical errors, such as sequencing error or artifacts during data handling that cause false prediction, we expect individual differences to be a major contributor for inconsistent genotype calls (<2%) between RNA-Seq and DNA-Seq data. In addition, monoallelic expression and RNA editing could be at least partially responsible for the observed inconsistency. In summary, although the gDNA of the F1 sample itself or its biological parents was not directly sequenced, we were able to establish high-confidence predicted F1 genotypes for further analyses based on the DNA-Seq data from gDNA pools.

### ASE in Embryonic Brain and Liver from F1 Cross Chickens

**Figure 2:**
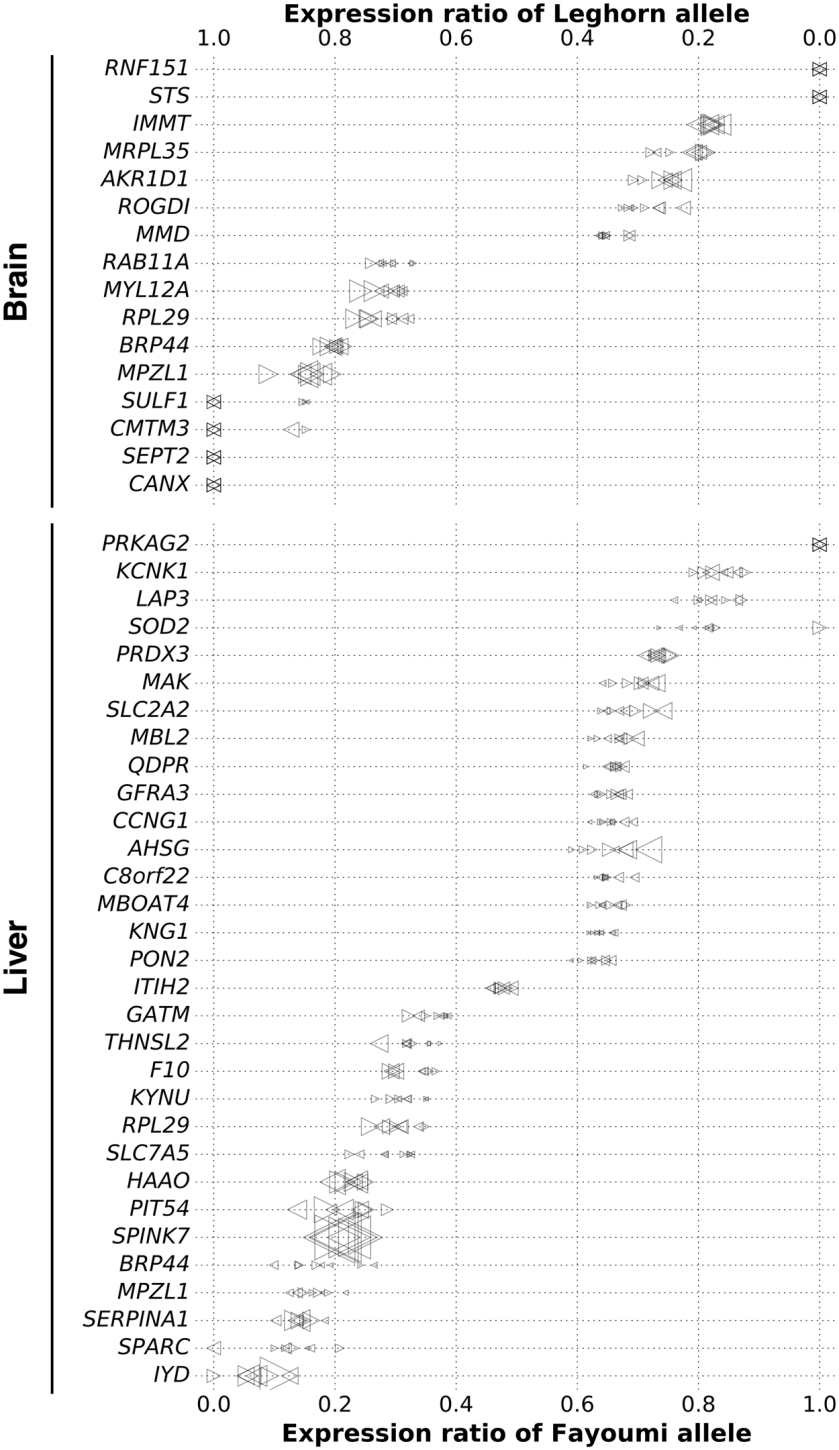
Partial list of the genes containing ASE SNPs observed in brain and liver of all samples. The upper X axis is the expression ratio of Leghorn allele and the lower X axis is the expression ratio of Fayoumi allele. Each dot represents a sample (Left triangle: FL cross; Right triangle: LF cross). The size of each marker is proportional to −log10(adjusted p-value).

We then examined ASE in embryonic brain and liver from Day 12 F1 embryos. On average, 51,935 and 30,336 loci were identified as being testable in brain and liver datasets per sample (Table S4), corresponding to 89.3% and 87.6% of the expressed genes in liver and brain, respectively. Analysis determined about 1.1% of testable loci in brain and 2.9% of testable loci in liver showing ASE pattern (Table S4), with a total of 2956 ASE SNPs in brain and 3436 ASE SNPs in liver. More than 99% of ASE observed in two or more samples showed higher expression towards the same alleles (Table S5). Particularly, ASE was detected for 52 SNPs in brain and 86 SNPs in liver in all samples from both crosses and with the same preferred alleles (Fig. 2; Table S5). Therefore, the preferred allele of those ASE was line-of-origin dependent. Moreover, the expression ratios of the preferred alleles (reads of preferred allele/total reads) were highly consistent across all the samples, as indicated by low coefficients of variation values (average CV = 3.7%; Table S6). Meta-analysis across samples identified 4,658 ASE SNPs in brain and 3,974 ASE SNPs in liver, and those ASE SNPs showed high overlapping with the results from within-sample analysis (Table S7). Combining the results from within- and across-sample analyses, we identified 5,181 ASE SNPs in brain and 4,617 ASE SNPs in liver.

Annotation of ASE SNPs led to the identification of 2,242 ASE genes in brain and 1,735 ASE genes in liver, which represented 18.3% of the expressed genes in Day 12 embryonic brain and 17.3% of the expressed genes in Day 12 embryonic liver. Next, we compared ASE genes in embryonic brain and liver to identify tissue-specific ASE. There were 832 ASE genes observed in both tissues, yet 1,454 (64.8%) ASE genes in brain and 947 (54.5%) ASE genes in liver were found to be tissue specific (Figure S2). Those ASE genes included 334 genes that were only expressed in brain and 148 genes that were only expressed in liver. Tissue-specific ASE has also been reported previously in human, mice, and cows^44–46^. Taken together, as in mammals, ASE is common in chicken embryonic brain and liver and shows tissue-specificity.

### Genome Imprinting is Absent in Day 12 Chicken Embryonic Brain and Liver

Based on ASE SNP discovery, we investigated the absence or existence of genomic imprinting in Day 12 chicken embryonic brain and liver. We took advantage of multiple biological replicates in our experiment and applied a set of criteria (see methods) to detect genes subjected to imprinting. As a proof of our approach, we identified SNPs in mitochondrial genes in both brain and liver meeting our criteria, and the expressed alleles of SNPs in mitochondrial genes were all of maternal origin, as expected. As for the nuclear genome, no SNPs in brain or in liver met our criteria.

The debate revolving around whether genomic imprinting exists in chickens has been going on for decades, and it has ignited several efforts to ascertain the answer to the question. RNA-Seq is emerging as an excellent tool for this purpose, as it surveys the global gene expression and therefore is able to examine the phenomenon of genomic imprinting at the genome-wide level. To date, most studies by examining gene expression have resulted in rejection of the existence of genomic imprinting in chickens. A well-known hypothesis of genomic imprinting, the “parent-conflict” theory, does not support the existence of genomic imprinting in egg-laying animals^47,48^. The theory suggests that genes from both parents are competing during embryonic development, with the paternally-derived genes promoting embryonic growth to facilitate the preservation of paternally-derived genes while maternally-derived genes limiting embryonic growth to conserve maternal resources for future offspring and for the fitness of maternally-derived genes. Hence, genomic imprinting should be present mostly in placental but not oviparous animals, as the maternal resource for the offspring is predetermined and the conflict of interest between parental alleles should not occur in oviparous animals. Supporting this hypothesis, the phenomenon of genomic imprinting has been found in eutherians and marsupials but not in monotremes, reptiles, amphibians and fish (summarized by Kaneda^49^). Collectively, our results and the results from Fresard *et al*. and Wang *et al*. studies^24,25^ suggest that genomic imprinting is missing in chickens, at least at the examined embryonic stages and early post-hatch. However, due to the complexity of gene regulation and the spatiotemporal nature of epigenetic modification, existence of genomic imprinting in other tissue types or developmental stages in chickens cannot be ruled out. Examining the DNA methylome profiles in reciprocal crosses could provide a new perspective on the conundrum of whether genomic imprinting exists in chickens. It is also important to note that sex differences may be present in ASE and genomic imprinting^50^. Future studies focusing on the female samples would be beneficial to comprehensively understand genomic imprinting in chickens.

### Genome-wide and Chromosome-wide Allelic Expression

**Figure 3:**
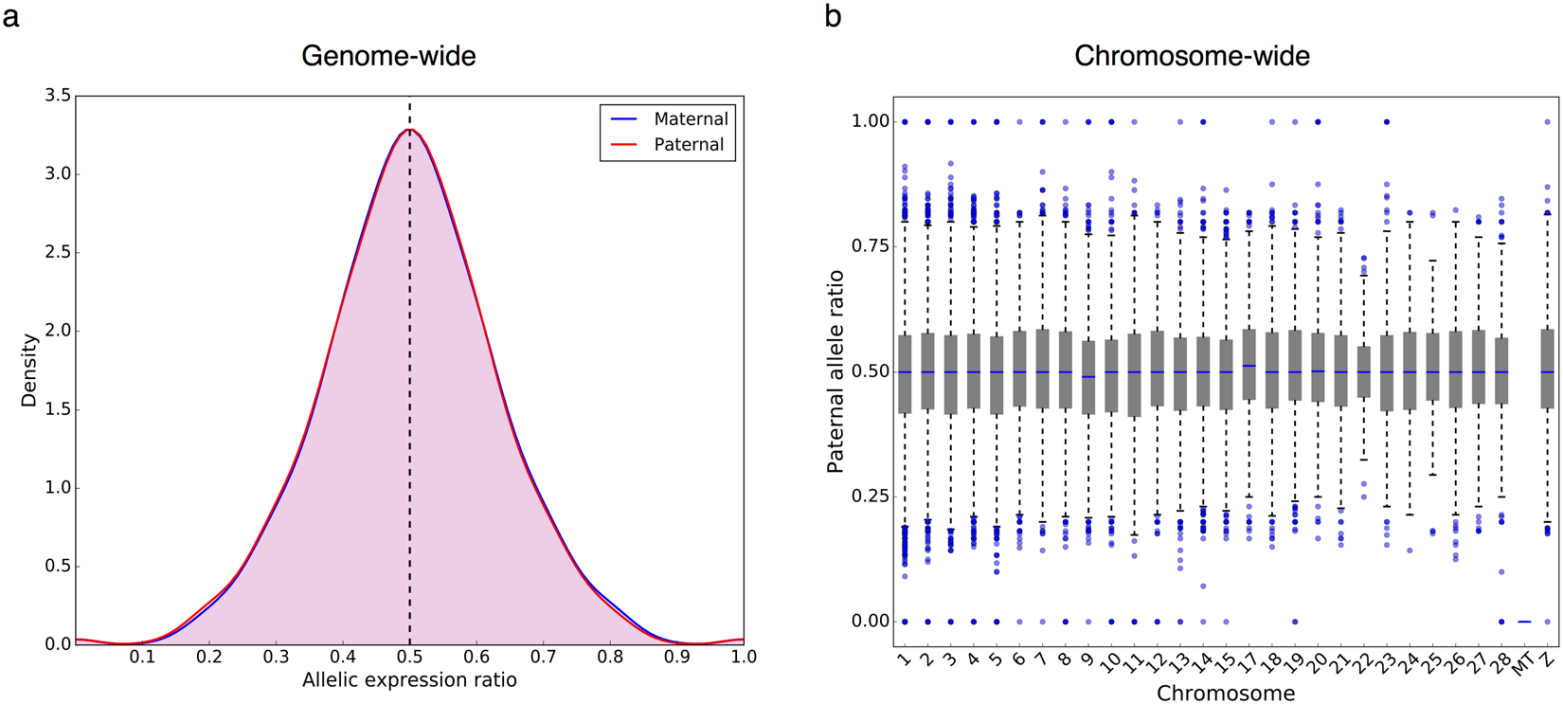
Balanced expression at genome-wide and chromosome-wide levels: (**a**) The distribution of paternal and maternal expression ratios at genome-wide level. (**b**) Box plot of paternal allele expression ratio for each chromosome.

Next, we queried the data to determine whether there is an imbalance of expression between paternal and maternal alleles at the genome-wide or chromosome-wide levels. We focused on the predicted heterozygous loci in F1 samples and calculated the expression ratios of paternal and maternal alleles (read counts of paternal allele or maternal allele/total read counts) for all samples. The genome-wide distribution of maternal and paternal allelic expression ratios did not show evident skewness (Fig. 3a). At the chromosome level, the median values of parental allelic ratio were approximately 0.5 (except for the mitochondrial chromosome) (Fig. 3b, Table S8). The results suggested that the expression of paternal and maternal alleles is roughly equal at genome-wide and chromosome-wide levels. Of note, in mice and cattle expression of the paternal alleles is favored^46,51^. In dairy cattle, 54.17% of ASE genes showed preference towards the paternal allele^46^. Our data of within-sample analysis suggested the percentages of ASE genes with higher expression of paternal allele ranged from 47.42% to 55.60%, with averages of 51.45% in brain and 50.91% in liver. Cowley *et al*. hypothesized that genome-wide imbalance between paternal and maternal alleles is prior to classic genomic imprinting in the evolutionary view, because natural selection may intensify the imbalance and develop it into more extreme cases – imprinting^51^. Based on our study, chickens lack genome-wide and chromosome-wide imprinting as well. Taken together, we demonstrated at multiple levels that genomic imprinting is absent in E12 chicken brain and liver. It is possible that the process of acquiring genomic imprinting did not occur in chickens.

### Dosage Compensation

Dosage compensation balances gene expression between females and males to compensate for the dosage difference caused by different copy numbers of sex chromosomes. Unlike humans and mice, birds are homogametic for males (ZZ) and heterogametic for females (ZW). Here we compared the mean of log-transformed FPKM values of the expressed genes between Z chromosome and autosomes and between sexes. The average values indicating the difference of gene expression between Z chromosomes and autosomes across all the samples were −0.34 (Z-to-A ratio 0.79) for brain and −0.38 (Z-to-A ratio 0.77) for liver. Additionally, we checked other available RNA-Seq data in our lab and found the difference of gene expression in breast muscle^52^, liver (unpublished) and abdominal fat^53^ from Day 47 post-hatch male chickens was −0.31 (Z-to-A ratio 0.81), −0.24 (Z-to-A ratio 0.85) and −0.41 (Z-to-A ratio 0.75), respectively. A recent experiment using both female and male breast muscle samples from our lab (unpublished) showed the expression difference of −0.17 (Z-to-A ratio 0.89) in males and −0.48 (Z-to-A ratio 0.72) in females. Z-linked genes were expressed lower than autosomal genes in all the tissues examined in the current study. Although previous studies of gene expression microarray and protein mass spectrometry showed the Z-to-A ratio close to 1 in males^54,55^, RNA-Seq data, including our results, suggested gene expression is not equalized between Z chromosome and autosomes in chickens^55,56^. After between-sample normalization, we found the male-to-female ratio for autosomal genes was 1.00, while the ratio for Z-linked gene expression was 1.35. This result corroborated previous reports that dosage is not fully compensated between females and males and that the Z-linked genes in male chickens express 1.2 to 1.6 times higher than that in female chickens^54,57,58^.

The mechanisms of dosage compensation in chickens are not fully understood. As previously mentioned, we found that the mean ratio of the paternal expression across all testable loci on the Z chromosome is approximately 0.5 (Fig. 3), indicating an almost equal expression of paternal and maternal alleles on Z chromosome. The results ruled out the possibility of imprinted chromosome inactivation as seen in marsupials, but it is still possible that some genes on paternal and maternal Z chromosomes are subjected to random inactivation. However, recent studies suggested Z chromosome inactivation is absent in chickens based on ASE analysis and correlation of allelic expressions between parents and crosses^56,59^. In fact, heterogeneity of cells due to possible random inactivation of some genes on paternal and maternal Z chromosomes may complicate examining Z-inactivation if RNA-Seq of tissue samples was performed. Future experiments using single cell sequencing may provide more direct evidence to demystify dosage compensation in chickens.

### Verification of ASE Using Sanger Sequencing

**Figure 4:**
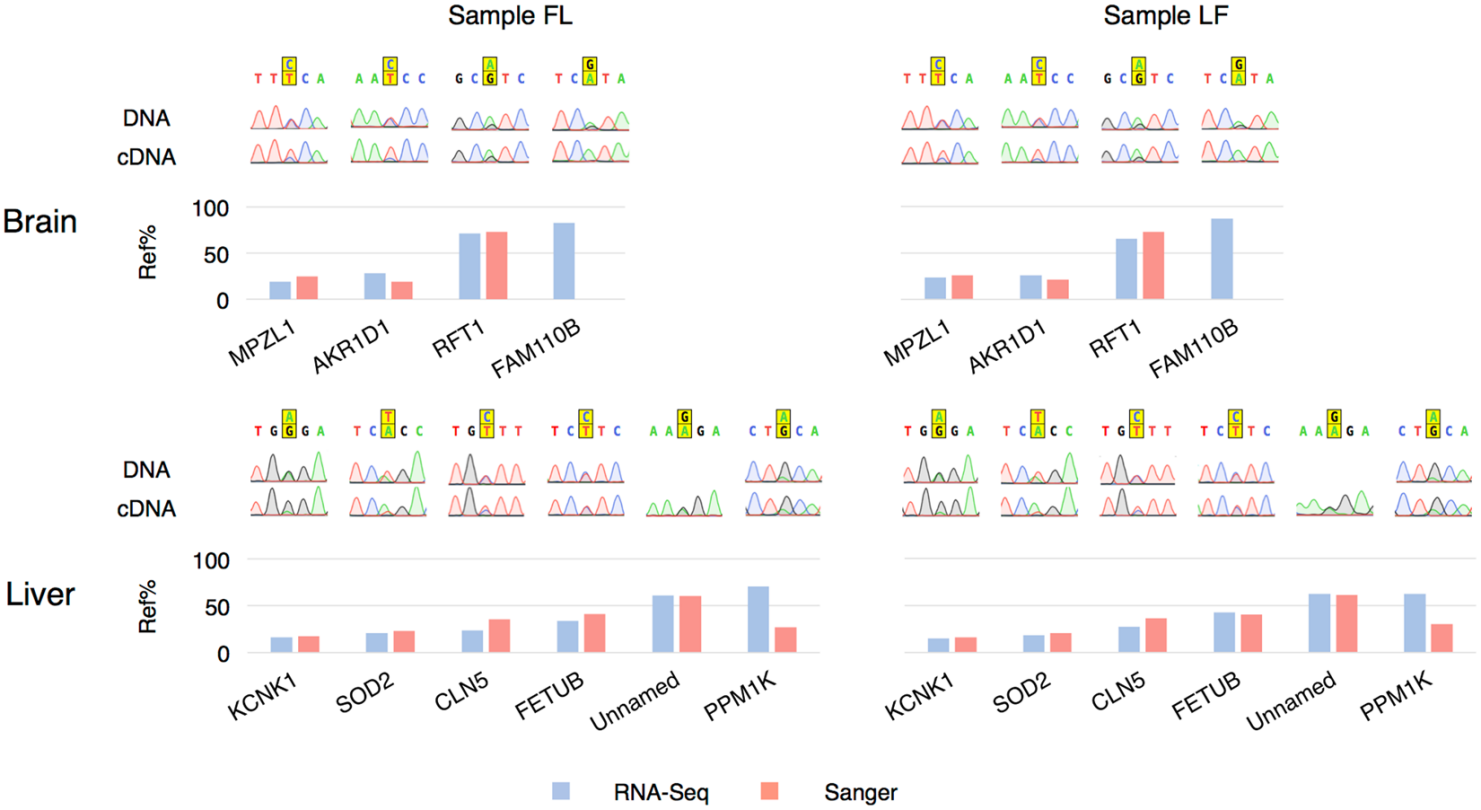
Verification of selected ASE genes by Sanger sequencing. The reference allele is displayed on top of the alternative allele at target heterozygous loci, and the barplot shows the expression ratios of the reference allele from RNA-Seq and Sanger Sequencing. Unnamed gene refers to gene ENSGALG00000027334.

To verify the results of ASE analysis from RNA-Seq data, we chose 10 ASE genes and examined the allelic expression using Sanger sequencing in one FL sample and one LF sample (Fig. 4). The expression of 4 genes was tested in brain samples and 6 genes in liver samples. The Sanger sequencing results of gDNA confirmed our prediction of heterozygous genotypes in both FL and LF samples. The cDNA was sequenced and analyzed to estimate the allelic ratios as described in other studies^60,61^. For eight out of the ten genes, the allelic ratios estimated by Minor Variant Finder agreed with the results from RNA-Seq. *FAM110B* in brain and *PPM1K* in liver showed different preferred alleles and allelic ratios in Sanger sequencing than RNA-Seq. Further examination found that the forward primer for *PPM1K* could only bind to two of the three transcript isoforms of the target region and thus may cause the discrepancy between RNA-Seq and Sanger sequencing. The cause for *FAM110B* discrepancies is unknown, but we speculate the primers for *FAM110B* were only able to amplify one transcript isoform as we only observed one peak in the chromatogram at the target SNP location.

## Conclusion

This study set out to investigate whether genomic imprinting exists in chickens by evaluating ASE. Different from previous RNA-Seq studies on genomic imprinting^24,25^, our study investigated two tissue types, brain and liver, from Day 12 male chicken embryos, with the aim of scrutinizing the absence or existence of genomic imprinting in chickens. The results showed no evidence for genomic imprinting in Day 12 embryonic brain or liver. Nonetheless, we identified thousands of ASE SNPs and tissue-specific ASE SNPs. We also identified some ASE SNPs with preferred alleles showing consistent line-of-origin effect in expression across multiple samples. We observed that the expression of paternal and maternal alleles is generally balanced at both genome-wide and chromosome-wide levels. Additionally, the gene expression on the Z chromosome is lower than that of the autosomes, and the dosage difference of sex chromosomes between female and male chickens is not fully compensated by gene expression in chickens. In the future, studies elucidating the relationship between ASE and economically important traits in chickens would be of great importance to fundamental studies as well as for practical application in poultry breeding.

## Electronic supplementary material

Supplementary Information

Supplementary Dataset 1

Supplementary Dataset 2
