# Supplementary material for: RNA-Seq Analyses Identify Frequent Allele Specific Expression and No Evidence of Genomic Imprinting in Specific Embryonic Tissues of Chicken
Source: Sci Rep. 2017 Sep 20;7:11944. doi: 10.1038/s41598-017-12179-9 (PMC5607270; doi:10.1038/s41598-017-12179-9)
Supplement: Supplementary file 1 — Supplementary Information [file 41598_2017_12179_MOESM1_ESM.pdf]

Supplementary Information

RNA-Seq Analyses Identify Frequent Allele Specific Expression and No Evidence of Genomic Imprinting in Specific Embryonic Tissues of Chicken

Zhu Zhuo [1], Susan J. Lamont [2], Behnam Abasht [1*]

[1] Department of Animal and Food Sciences, University of Delaware

[2] Department of Animal Science, Iowa State University

* Corresponding author: Email: abasht@udel.edu

Figure S1 PCR-based sexing

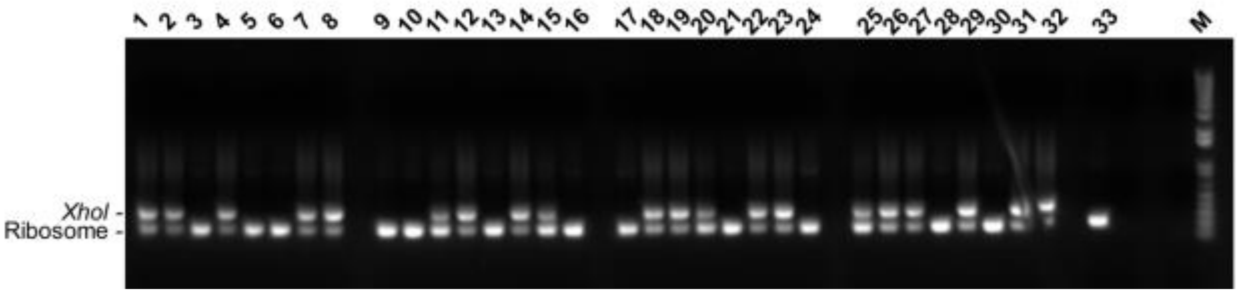

Figure S2 Venn diagram of tissue-specific ASE SNPs

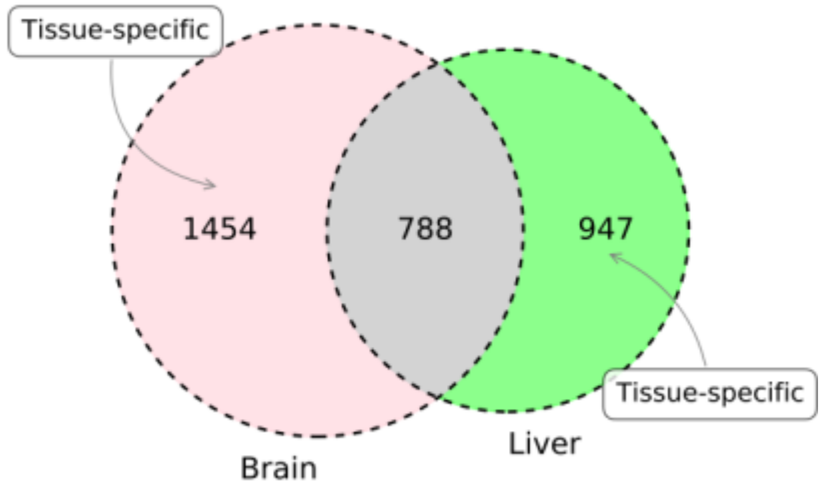

Table S1 Results of PCR-based sexing

	Dam ID	Sample ID	line / cross *	Sex	Note
1	1379	126	FL	F	
2	1380	127	FL	F	
3	1378	118	FL	M	
4	1379	124	FL	F	
5	1379	123	FL	M	Included in RNA-Seq
6	1367	106	LF	M	Included in RNA-Seq
7	1769	142	L	F	
8	1379	122	FL	F	
9	1367	105	LF	M	Included in RNA-Seq
10	1769	141	L	M	
11	1784	152	F	F	
12	1379	125	FL	F	
13	1367	104	LF	M	Included in RNA-Seq
14	1769	140	L	F	
15	1784	151	F	F	
16	1379	121	FL	M	Included in RNA-Seq
17	1784	150	F	M	Included in RNA-Seq
18	1774	146	L	F	
19	1380	131	FL	F	
20	1367	103	LF	F	
21	1784	149	F	M	Included in RNA-Seq
22	1774	145	L	F	
23	1380	130	FL	F	
24	1367	102	LF	M	Included in RNA-Seq
25	1790	153	F	F	
26	1774	144	L	F	
27	1380	128	FL	F	
28	1766	136	L	M	Included in RNA-Seq
29	1372	113	FL	F	
30	1378	115	FL	M	Included in RNA-Seq
31	1768	137	L	F	Included in RNA-Seq
32	1768	138	L	F	
33	1378	117	FL	M	Included in RNA-Seq

* F: inbred Fayoumi line
L: inbred Leghorn line
FL: Fayoumi (father) and Leghorn (mother) cross
LF: Leghorn (father) and Fayoumi (mother) cross

Table S2 Statistics of DNA-Seq data

Sample	Total Reads	Coverage	Mapped	Paired	Single Nucleotide Variants *		
					Total	Homozygous	Heterozygous
Fayoumi	155,828,716	21.8 X	98.59%	92.37%	4,442,390	4,158,536	283,854
Leghorn	137,444,974	19.2 X	98.46%	92.31%	4,055,489	3,886,221	169,268

*Variants relative to the chicken reference genome

Table S3 Statistics of RNA-Seq data

Sample	Total Reads	Mapping Rate	
		Ori. Ref.	Cus. Ref.
FF_BR_1784_149	55,287,524	84.23%	84.38%
FF_BR_1784_150	58,368,384	85.09%	85.29%
FF_LV_1784_149	65,150,556	87.06%	87.10%
FF_LV_1784_150	54,968,474	88.28%	88.32%
FL_BR_1378_115	55,494,754	80.92%	81.08%
FL_BR_1378_117	69,508,092	79.15%	79.37%
FL_BR_1379_121	71,864,328	80.17%	80.42%
FL_BR_1379_123	59,828,718	80.44%	80.70%
FL_LV_1378_115	71,221,304	88.50%	88.55%
FL_LV_1378_117	58,659,416	86.05%	86.09%
FL_LV_1379_121	70,418,648	86.84%	86.88%
FL_LV_1379_123	57,658,364	82.98%	83.01%
LF_BR_1367_102	70,756,508	85.92%	86.18%
LF_BR_1367_104	71,414,926	80.80%	81.00%
LF_BR_1367_105	71,053,346	85.27%	85.48%
LF_BR_1367_106	57,530,806	79.93%	80.11%
LF_LV_1367_102	67,539,336	83.36%	83.39%
LF_LV_1367_104	68,672,610	86.14%	86.17%
LF_LV_1367_105	72,362,228	85.76%	85.82%
LF_LV_1367_106	50,747,202	85.89%	85.92%
LL_BR_1766_136	74,012,630	87.33%	87.46%
LL_BR_1769_141	66,180,806	83.25%	83.44%
LL_LV_1766_136	72,089,856	86.23%	86.27%
LL_LV_1769_141	64,525,424	88.70%	88.73%

Ori.Ref.: Results from original chicken reference genome
Cus.Ref.: Results from customized reference genome

Table S4 Number of testable loci and ASE SNPs in each sample

Sample	No. of testable loci	No of ASE SNPs	No. of ASE SNPs with higher expression of paternal allele	No. of ASE SNPs with higher expression of maternal allele	No. of ASE SNPs with higher expression of reference allele	No. of ASE SNPs with higher expression of alternative allele
FL_BR_1378_115	52651	463	214	249	246	217
FL_BR_1378_117	56314	590	295	295	315	275
FL_BR_1379_121	51099	605	291	314	328	277
FL_BR_1379_123	46742	422	201	221	234	188
LF_BR_1367_102	45020	619	333	286	324	295
LF_BR_1367_104	55961	782	464	318	423	359
LF_BR_1367_105	52587	699	373	326	385	314
LF_BR_1367_106	55108	484	255	229	247	237
Average	51935	583				
FL_LV_1378_115	28247	936	481	455	461	475
FL_LV_1378_117	25970	893	446	447	434	459
FL_LV_1379_121	34616	1119	578	541	557	562
FL_LV_1379_123	26845	600	300	300	303	297
LF_LV_1367_102	33675	1061	544	517	536	525
LF_LV_1367_104	33208	915	504	411	445	470
LF_LV_1367_105	33263	904	447	457	459	445
LF_LV_1367_106	26861	649	338	311	309	340
Average	30336	885				

Table S5 Number of ASE SNPs observed in 1 or more samples and consistency of preferred alleles

	Brain		Liver	
Observed in # of samples	No. of ASE SNPs	No. of ASE SNPs with consistent preferred allele	No. of ASE SNPs	No. of ASE SNPs with consistent preferred allele
1	2267	Not Applicable	2156	Not Applicable
2	326	325	467	460
3	131	131	234	232
4	68	67	169	168
5	44	44	116	116
6	32	32	115	114
7	36	36	93	93
8	52	50*	86	84*

*The two inconsistent SNPs are located at mitochondria DNA, so the preferred allele is always maternal

Table S6 CV and mean expression ratio of the preferred allele for the ASE SNPs identified in all samples

Chromosome	Position	Reference	Average expression ratio of preferred allele	SD	CV
Brain					
1	56721704	C	0.73	0.04	5.0%
1	56731039	G	0.75	0.03	3.6%
1	90871453	A	0.79	0.04	4.8%
1	90871581	T	0.78	0.03	3.4%
1	90871607	C	0.77	0.03	4.6%
1	90871693	T	0.75	0.05	6.3%
1	90871813	C	0.79	0.04	5.1%
1	90871952	G	0.80	0.05	6.5%
1	90872023	A	0.78	0.04	5.4%
1	90872062	A	0.76	0.05	5.9%
1	90872148	T	0.78	0.04	5.6%
1	90872238	C	0.77	0.03	3.4%
1	90872296	T	0.79	0.05	6.4%
1	90872302	A	0.80	0.05	6.0%
1	90872431	T	0.81	0.04	5.2%
1	90872458	T	0.81	0.03	4.3%
1	90872516	C	0.82	0.03	3.9%
1	90872557	A	0.82	0.04	4.6%
1	90872709	T	0.80	0.04	5.1%
1	90872734	A	0.82	0.03	3.9%
1	90872872	G	0.82	0.03	3.3%
1	90873011	G	0.86	0.03	3.4%
1	90873038	T	0.85	0.02	2.7%
1	90873438	G	0.82	0.04	4.9%
1	90873705	C	0.82	0.04	5.1%
1	90873752	A	0.83	0.02	2.6%
1	90875332	G	0.85	0.02	2.1%
1	90875606	A	0.86	0.06	6.9%
1	90891019	G	0.83	0.04	4.6%
1	98653919	G	0.67	0.03	5.1%
1	125763437	C	1.00	0.00	0.0%
2	100545252	C	0.71	0.02	3.4%
2	115993458	G	0.94	0.08	8.3%
2	125910349	A	1.00	0.00	0.0%
3	45157492	T	0.79	0.03	4.2%

4	13273040	A	0.77	0.02	2.4%
4	45797087	A	0.71	0.04	5.8%
4	85062164	A	0.82	0.04	5.0%
4	85062616	A	0.81	0.02	2.7%
4	85063328	G	0.84	0.02	2.5%
4	85064009	G	0.81	0.05	5.7%
4	85064068	C	0.78	0.04	4.7%
9	4497393	T	1.00	0.00	0.0%
10	17662128	A	0.71	0.02	3.5%
11	10904781	T	0.96	0.07	6.8%
12	532343	A	0.72	0.03	4.3%
13	12857392	A	1.00	0.00	0.0%
13	12857409	G	1.00	0.00	0.0%
14	14415138	G	0.71	0.03	4.8%
18	5920301	C	0.65	0.02	3.2%
MT	2207	C	1.00	0.00	0.0%
MT	8058	G	1.00	0.00	0.0%

Liver					
1	4110771	T	0.62	0.02	3.7%
1	4120158	C	0.62	0.02	4.0%
1	4121228	C	0.98	0.06	6.0%
1	4126505	T	0.64	0.03	4.1%
1	90872709	T	0.81	0.05	6.5%
1	90872734	A	0.83	0.06	7.0%
1	90891019	G	0.83	0.03	3.6%
1	136923134	C	0.68	0.03	4.7%
1	154785520	C	1.00	0.00	0.0%
1	191988736	C	1.00	0.00	0.0%
2	6378773	A	1.00	0.00	0.0%
2	6380603	C	1.00	0.00	0.0%
2	6380618	G	1.00	0.00	0.0%
2	6380661	A	1.00	0.00	0.0%
2	6380732	G	1.00	0.00	0.0%
2	6380790	G	1.00	0.00	0.0%
2	6381377	C	1.00	0.00	0.0%
2	6382777	T	1.00	0.00	0.0%
2	6382834	A	1.00	0.00	0.0%
2	6382944	C	1.00	0.00	0.0%
2	6383417	T	1.00	0.00	0.0%
2	6383419	C	1.00	0.00	0.0%
2	7817808	C	1.00	0.00	0.0%
2	7817888	C	1.00	0.00	0.0%

2	23986930	A	0.63	0.02	3.6%
2	63336244	A	0.69	0.03	4.5%
2	108191863	C	0.65	0.02	3.1%
3	23462744	T	0.77	0.03	4.1%
3	23462966	C	0.77	0.02	3.0%
3	23462969	T	0.77	0.02	2.9%
3	38008398	T	0.84	0.07	8.0%
3	38008628	T	0.85	0.07	8.1%
3	38009070	A	0.83	0.04	4.9%
3	38009219	T	0.82	0.03	3.6%
3	41123224	T	0.76	0.05	6.4%
3	44930274	T	0.82	0.08	9.5%
3	47729536	T	0.89	0.05	5.9%
3	47730778	A	0.97	0.05	5.2%
4	49039601	G	0.66	0.02	2.8%
4	49039636	G	0.65	0.03	4.4%
4	59600455	G	0.61	0.02	3.8%
4	75553631	G	0.82	0.04	4.5%
4	75582961	A	0.66	0.02	3.0%
4	85518541	T	0.68	0.06	9.0%
4	85519658	A	0.66	0.03	4.7%
5	17675701	G	0.77	0.05	6.1%
5	44822999	T	0.85	0.03	3.2%
5	44823536	G	0.85	0.04	4.4%
5	44824890	C	0.87	0.05	6.2%
5	44825474	T	0.85	0.02	2.9%
6	3981946	T	0.66	0.03	3.9%
6	29560427	C	0.74	0.05	6.5%
6	29560651	C	0.71	0.03	4.2%
6	29560766	A	0.74	0.02	2.1%
6	29560788	A	0.74	0.02	2.0%
6	29560923	T	0.73	0.03	4.2%
6	29560959	T	0.74	0.03	4.1%
7	32197070	T	0.69	0.03	4.1%
9	8999054	C	1.00	0.00	0.0%
9	15612772	C	0.65	0.04	6.5%
9	15659017	A	0.64	0.01	2.3%
9	19180724	G	0.68	0.04	5.3%
10	10413182	C	0.64	0.02	3.8%
11	17464533	T	0.73	0.04	6.0%
11	17465161	G	0.69	0.04	6.4%
12	532343	A	0.70	0.03	4.3%
12	14640323	C	0.63	0.02	3.0%

12	14640486	T	0.69	0.03	4.6%
13	6078755	G	0.67	0.02	3.4%
13	6079944	A	0.63	0.02	3.1%
13	6081530	C	0.67	0.04	5.8%
13	9877048	C	0.79	0.02	3.0%
13	9885038	G	0.78	0.03	3.9%
13	9885062	T	0.78	0.03	3.2%
13	12246793	G	0.88	0.06	6.9%
13	17577272	T	0.66	0.02	3.0%
15	6211426	C	0.69	0.02	2.6%
15	6211517	C	0.73	0.03	4.7%
15	8105432	C	0.61	0.02	3.4%
16	310634	G	0.98	0.06	6.6%
28	794340	T	0.69	0.04	6.4%
LGE64	182232	G	1.00	0.00	0.0%
LGE64	547553	C	0.77	0.06	7.1%
LGE64	548701	C	0.79	0.04	5.0%
MT	2207	C	1.00	0.00	0.0%
MT	8058	G	1.00	0.00	0.0%

Table S7 Number of ASE SNPs and genes identified in within-sample and across-sample (meta-analysis) analyses using F1 samples

	Meta-analysis (adjust p-value < 0.05)	Within-sample analysis (adjust p-value < 0.1)	Overlapping	Total
Brain				
ASE SNPs	4658	2952	2429	5181
ASE Genes	1998	1523	1279	2242
Liver				
ASE SNPs	3974	3423	2780	4617
ASE Genes	1455	1422	1142	1735

Table S8 Median of paternal expression ratio per chromosome

Sample/chromosome	1	2	3	4	5	6	7	8	9	10	11	12	13	14	15	17	18	19	20	21	22	23	24	25	26	27	28	MT	Z
FL_BR_1378_115	0.50	0.50	0.50	0.50	0.50	0.50	0.50	0.50	0.49	0.50	0.50	0.50	0.50	0.50	0.50	0.51	0.50	0.50	0.50	0.50	0.50	0.50	0.50	0.50	0.50	0.50	0.50	0.00	0.50
FL_BR_1378_117	0.50	0.50	0.50	0.50	0.50	0.50	0.50	0.50	0.50	0.50	0.50	0.50	0.50	0.50	0.50	0.50	0.50	0.50	0.50	0.50	0.51	0.50	0.50	0.50	0.50	0.51	0.51	0.00	0.50
FL_BR_1379_121	0.50	0.50	0.50	0.50	0.50	0.50	0.50	0.50	0.50	0.50	0.50	0.50	0.50	0.50	0.50	0.51	0.51	0.50	0.51	0.50	0.50	0.50	0.52	0.49	0.52	0.51	0.51	0.00	0.50
FL_BR_1379_123	0.50	0.50	0.50	0.50	0.50	0.50	0.50	0.50	0.50	0.50	0.50	0.50	0.50	0.51	0.50	0.50	0.50	0.50	0.51	0.51	0.51	0.51	0.50	0.51	0.51	0.50	0.50	0.00	0.50
FL_LV_1378_115	0.50	0.50	0.50	0.50	0.50	0.50	0.50	0.50	0.50	0.50	0.50	0.50	0.52	0.50	0.49	0.50	0.53	0.50	0.52	0.50	0.51	0.51	0.50	0.50	0.50	0.47	0.51	0.00	0.50
FL_LV_1378_117	0.50	0.50	0.50	0.50	0.50	0.51	0.50	0.50	0.50	0.49	0.50	0.49	0.50	0.50	0.50	0.50	0.51	0.51	0.49	0.50	0.50	0.50	0.50	0.47	0.48	0.50	0.52	0.00	0.49
FL_LV_1379_121	0.50	0.50	0.50	0.50	0.50	0.51	0.50	0.50	0.50	0.50	0.50	0.50	0.50	0.50	0.50	0.51	0.50	0.50	0.50	0.48	0.50	0.50	0.52	0.47	0.50	0.50	0.50	0.00	0.50
FL_LV_1379_123	0.50	0.50	0.50	0.50	0.50	0.51	0.50	0.50	0.50	0.50	0.50	0.49	0.50	0.50	0.50	0.50	0.50	0.50	0.50	0.49	0.50	0.51	0.50	0.45	0.50	0.48	0.50	0.00	0.50
LF_BR_1367_102	0.50	0.50	0.50	0.50	0.50	0.50	0.50	0.50	0.50	0.50	0.50	0.50	0.50	0.50	0.51	0.50	0.50	0.51	0.49	0.51	0.50	0.48	0.51	0.49	0.50	0.50	0.50	0.00	0.50
LF_BR_1367_104	0.50	0.50	0.50	0.50	0.50	0.50	0.50	0.50	0.50	0.50	0.50	0.50	0.50	0.50	0.50	0.50	0.49	0.50	0.50	0.49	0.49	0.49	0.50	0.52	0.50	0.49	0.49	0.00	0.50
LF_BR_1367_105	0.50	0.50	0.50	0.50	0.50	0.50	0.50	0.50	0.50	0.50	0.50	0.50	0.50	0.50	0.51	0.50	0.50	0.50	0.50	0.50	0.50	0.50	0.51	0.47	0.50	0.48	0.50	0.00	0.50
LF_BR_1367_106	0.50	0.50	0.50	0.50	0.50	0.50	0.50	0.50	0.50	0.50	0.50	0.50	0.50	0.50	0.50	0.50	0.50	0.50	0.50	0.49	0.49	0.50	0.50	0.53	0.49	0.50	0.49	0.00	0.50
LF_LV_1367_102	0.50	0.50	0.50	0.50	0.50	0.49	0.50	0.50	0.50	0.50	0.50	0.50	0.50	0.50	0.50	0.50	0.50	0.50	0.50	0.52	0.50	0.49	0.49	0.53	0.51	0.54	0.49	0.00	0.50
LF_LV_1367_104	0.50	0.50	0.50	0.50	0.50	0.50	0.50	0.50	0.50	0.50	0.50	0.50	0.50	0.50	0.50	0.50	0.49	0.49	0.50	0.51	0.49	0.49	0.50	0.50	0.52	0.50	0.50	0.00	0.50
LF_LV_1367_105	0.50	0.50	0.50	0.50	0.50	0.49	0.50	0.50	0.50	0.50	0.50	0.50	0.50	0.50	0.50	0.50	0.49	0.50	0.50	0.50	0.51	0.50	0.49	0.50	0.49	0.52	0.50	0.00	0.50
LF_LV_1367_106	0.50	0.50	0.50	0.50	0.50	0.50	0.50	0.50	0.50	0.50	0.50	0.50	0.50	0.50	0.51	0.50	0.50	0.50	0.50	0.52	0.52	0.49	0.50	0.50	0.53	0.52	0.50	0.00	0.50
Average	0.50	0.50	0.50	0.50	0.50	0.50	0.50	0.50	0.50	0.50	0.50	0.50	0.50	0.50	0.50	0.50	0.50	0.50	0.50	0.50	0.50	0.50	0.50	0.50	0.50	0.50	0.50	0.00	0.50
